# COVID-19, Economic Impact, Mental Health, and Coping Behaviors: A Conceptual Framework and Future Research Directions

**DOI:** 10.3389/fpsyg.2021.759974

**Published:** 2021-11-11

**Authors:** Xiaoqian Lu, Zhibin Lin

**Affiliations:** ^1^School of Business Administration, Jimei University, Xiamen, China; ^2^Durham University Business School, Durham University, Durham, United Kingdom

**Keywords:** COVID-19 pandemic, economic difficulty, employment difficulty, mental health, coping behavior

## Abstract

The COVID-19 pandemic has caused serious economic and social consequences. Recent research shows that the pandemic has not only caused a physical health crisis but also caused many psychological and mental crises. Based on the contemporary cognitive-behavioral models, this article offers a conceptual analysis of how the pandemic affects individual mental health and coping behaviors from the perspective of individual economic status, individual context, and social context. The analysis shows that (1) the pandemic has led to increased economic uncertainty, increased unemployment and underemployment pressure, increased income uncertainty, and different degrees of employment pressure and economic difficulties; (2) these difficulties have stimulated different levels of mental health problems, ranging from perceived insecurity (environmental, food safety, etc.), worry, fear, to stress, anxiety, depression, etc., and the mental health deterioration varies across different groups, with the symptoms of psychological distress are more obvious among disadvantageous groups; and (3) mental health problems have caused behavior changes, and various stress behaviors such as protective behaviors and resistive behaviors. Future research directions are suggested.

## Introduction

The current COVID-19 pandemic is still ongoing, and it is concerning that we still do not know how long it will last and what long-term effects it will have. Despite the successful development of vaccines, the medical capacity to completely treat this disease is still limited. Non-pharmaceutical interventions (NPIs), such as increasing handwashing, reducing physical contact, wearing masks in public places, maintaining social distance, quarantine, and isolation, are still the main strategies for handling this pandemic (Van Bavel et al., 2020; Gössling et al., 2021). The social and economic consequences of the pandemic are devastating: almost half of the global workforce is at risk of losing their livelihoods, tens of millions are at risk of falling into extreme poverty, and millions of companies are facing existential threat (Alauddin et al., 2021). In addition to the pandemic itself, the economic impact of the crisis brings heavy psychological stress to individuals, causing mental health problems, and may trigger long-lasting behavior changes. Other pandemic-related factors may also cause psychological distress, including mandatory use of face masks (Wang et al., 2020a), lockdowns (Le et al., 2020), lack of access to medical services (Hao et al., 2020; Tee et al., 2021), dissatisfaction with health information (Tee et al., 2021), perceived discrimination (Wang et al., 2021), and stress about returning to work (Tan et al., 2020).

Prior behavioral science research focuses on perceived threats, stress, and coping (Van Bavel et al., 2020). In the early stages of the pandemic, the physical health risks associated with the COVID-19 pandemic have received extensive attention from the academic community (Mehta et al., 2020; Odayar et al., 2020), and there is growing research attention on the risks of mental health associated with the spread of the pandemic (Auerbach and Miller, 2020; Xiong et al., 2020; Wang et al., 2020a). The focal attention since the outbreak of the pandemic has been the psychological distress as a result of the pandemic itself (Jungmann and Witthöft, 2020) or the adverse economic impact of the pandemic (Bierman et al., 2021). However, it is still unclear how the pandemic control measures cause mental health problems through economic impact (Murakami et al., 2021). Many scholars believe that the measures taken during the pandemic may cause people to suffer more economic losses and fall into economic difficulties, thereby causing serious mental health problems (Timming et al., 2021), while some scholars believe that although the pandemic may cause huge economic losses, people’s mental health status has not decreased (Murakami et al., 2021). Therefore, it is necessary to conduct a conceptual analysis of the economic impact of the pandemic and mental health by synthesizing the relevant findings in existing literature (Ali et al., 2021).

This study aims to develop a conceptual framework linking the pandemic to individual economic problems, unemployment, mental health, and behavior change. The main research questions are (1) what kind of individual economic stress has the pandemic caused? 2) what mental health problems have this individual economic stress caused, and to what extent? 3) does the mental health problem vary by different groups or individuals? 4) what kind of behaviors may be caused by the deterioration of mental health?

## Theoretical Background

According to the World Health Organization, mental health includes subjective well-being, self-efficacy, autonomy, ability, intergenerational dependence, intellectual or emotional potential. When there is a problem with mental health, there will be a decline in subjective well-being and various negative emotions (such as fear, nervousness, loneliness, and despair), and symptoms such as mental distress (such as anxiety, depression, and stress) will appear (Hossain et al., 2020). Mental health issues are considered as public health problems that are often affected by factors related to occupation, employment opportunities, and economic stress (Ali et al., 2021). Many scholars have examined the impact of economic poverty and unemployment on mental health (Jin et al., 1997). Disaster mental health research also shows that people generally suffer emotional or psychological distress following a disaster (Pfefferbaum and North, 2020).

### Mental Health Amid the Pandemic

The World Health Organization (2020) proposes mental health indicators for the COVID-19 pandemic: painful symptoms and perceived danger. Mental distress is a short-term state of emotional distress, often driven by limited resources to manage stressors and daily life needs (Patel and Rietveld, 2020). The pandemic can become a major source of stress, especially in chronic anxiety and financial stress (Van Bavel et al., 2020). Mental distress has become the focus of research on mental health problems amid a large-scale crisis (Cheng et al., 2004; Wang et al., 2020b). Preliminary evidence suggests that symptoms of anxiety, depression, and self-reported stress are common psychological responses to the pandemic (Rajkumar, 2020). Salari et al. (2020) reported that the prevalence of stress was between 29.6 and 33.7%. In addition to mental distress, the pandemic and corresponding interventions or preventive measures may make people feel insecure, fearful, uncertain, lonely, or isolated (Auerbach and Miller, 2020), which exacerbates the psychological distress (Pfefferbaum and North, 2020).

### Public Health Interventions

Non-medical interventions or control measures during the pandemic may weaken social relationships that can help people to regulate emotions, cope with stress, and maintain adaptability (Rimé, 2009; Jetten et al., 2017; Williams et al., 2018), exacerbate feelings of loneliness and isolation (Hawkley and Cacioppo, 2010; Holmes et al., 2020), and become a risk factor for more serious mental health disorders (Cacioppo et al., 2006). The stresses experienced during the pandemic, especially the economic stress, may cause difficulties in interpersonal relationships, destroy psychological resources, and make normal interactions difficult (Karney, 2020). The impact of the pandemic interventions on mental health vary across different (employment) groups.

### Contemporary Cognitive-Behavioral Models and Mental Health

The contemporary cognitive-behavioral models (Taylor and Asmundson, 2004; Asmundson et al., 2010) explore the key role of traits, triggering events, cognition, and behaviors in the development and maintenance of health anxiety, which can be used to analyze mental health problems during the pandemic period. Jungmann and Witthöft (2020) believe that during the pandemic, idiosyncratic health anxiety regulates the relationship between excessive online information search and viral anxiety, and adaptive emotions serve as a buffer between the two. The “Role Tension” model explores mental health issues from the perspective of role conflicts. It believes that individuals with multiple social roles may experience role conflicts, resulting in stress and adverse mental health (Oomens et al., 2007). The broader behavioral immune system theory (McKay et al., 2020) explores the specific path of disease anxiety, and believes that disgust tendency and sensitivity, and emotional response are all part of the behavioral immune system.

## Conceptual Framework

**Figure 1:**
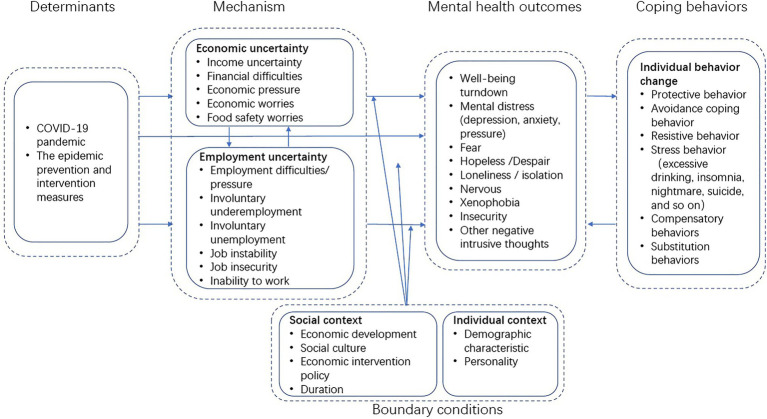
Conceptual framework.

Figure 1 summarizes the themes from recent research findings in a conceptual framework.

### The Mechanism of COVID-19’s Impact on Mental Health

In addition to the pandemic itself, the lockdown, quarantine, or self-isolation policies that aim at fighting the pandemic, the involuntary underemployment or unemployment have led to individuals’ economic difficulties and mental health problems of varying degrees for many people. The economic impact on individuals seems to further exuberate the suffering from the pandemic (Bierman et al., 2021).

#### Employment Uncertainty

Employment problems caused by the pandemic include involuntary unemployment (Piltch-Loeb et al., 2021), involuntary underemployment (Pierce et al., 2020; Ferry et al., 2021), employment uncertainty and insecurity (Wilson et al., 2020), job instability or inability to work (Sirviö et al., 2012), and others. Studies have shown that involuntary underemployment and/or unemployment are related to poor mental health (Dooley et al., 2000; Pharr et al., 2012), especially those who are unemployed during economic crises or recessions (Uutela, 2010; Drydakis, 2015; Fiori et al., 2016). It is reported that crisis-related unemployment has led to a sharp rise in psychological disorders in low- and middle-income countries (Uutela, 2010). Despite the government measures to limit the economic impact, the involuntary underemployment or unemployment caused by the epidemic is prominent.

The impact of the pandemic on mental health varies (Pierce et al., 2020). Long-term unemployed people are most vulnerable to adverse mental health effects (Pierce et al., 2020), and those who were employed and retired in the months before the pandemic experience worse than expected mental health conditions (Ferry et al., 2021). Reduced work has different effects on the mental health of different groups. People who are in a poor health condition or self-isolated, and those who have their work reduced due to care responsibilities, have a higher degree of psychological distress (Ferry et al., 2021). The higher the work insecurity caused by the pandemic, the more severe the symptoms of depression (Wilson et al., 2020). As the pandemic continues, the fear of the pandemic itself has not increased mental health problems, but the deterioration of the labor market and the increase in the unemployment rate may intensify people’s fear of unemployment, thereby increasing the degree of mental distress (Timming et al., 2021). In addition, due to the lockdown, people’s work routines can be broken. Remote work, interruption of work activities due to lockdown measures, or increased workload due to the needs of the pandemic may also become factors affecting mental health (Rossi et al., 2020).

#### Economic Uncertainty

The analysis of individual economic distress during the pandemic usually focus on short-term economic distress or economic stress, such as personal income uncertainty, personal financial difficulties, salary reduction and other economic (income) losses (Piltch-Loeb et al., 2021), as well as the expected long-term financial impact, such as depletion of savings and/or retirement funds (Piltch-Loeb et al., 2021).

There are two possible ways in which economic distress mediates the impact of the pandemic on psychological distress. One is the economic hardship or economic threat triggered by the pandemic itself. Individual economic loss, economic hardship, or economic threat was significantly associated with mental health (Ali et al., 2021). The pandemic has led to increased risks of depression, anxiety, stress, despair (Pettinicchio et al., 2021), insomnia (Hossain and Ali, 2021), and other common mental health problems. The negative relationship between economic distress and mental health may be a cumulative process. As exposure to distress extends, the average level of individual sufferings increases (Bierman et al., 2021). At the later stage in the pandemic, economic-related anxiety may be a major predictor of psychological distress (Timming et al., 2021).

Second, the unemployment and employment transition triggered by the pandemic affects the financial situation, which in turn affects psychological distress (Thomas et al., 2007). The economic recession triggered by the pandemic and the increase in economic uncertainty has led to business bankruptcy or downsizing, increased involuntary underemployment or unemployment, increased uncertainty in personal income, and increased likelihood of individuals or families experiencing financial difficulties and economic pressure, consequently triggering large scale mental health problems (Kimhi et al., 2020).

### Coping Behaviors

The direct consequence of the pandemic’s impact on mental health is the change of personal behavior and habits. Studies on past epidemics and pandemics have shown that negative emotions such as anxiety and stress during the epidemic may lead to different behavior patterns.

#### Positive Defensive Behavior

Humans are born with a set of defense systems against ecological threats (Mobbs et al., 2015). The main emotional response during a pandemic is fear. When people feel capable of responding to the threat of the pandemic, fear can cause individual behavior changes, but if people feel powerless, a defensive response occurs. Positive defensive behavior includes protective, defensive (avoidance), and substitution behaviors.

##### Protective Behavior

Mental health problems, such as high anxiety, during the epidemic may produce protective behaviors or compensatory behaviors (Wheaton et al., 2012), including washing hands frequently, wearing masks, increasing cleaning of items, social distancing, and other restrictions. Protective behavior can be voluntary (Rubin et al., 2009) or compliant with government regulation (Fragkaki et al., 2021). In addition, people actively engage in physical activities to cope with stress and anxiety (Ai et al., 2021).

##### Defensive (Avoidance) Behavior

Such behavior includes avoiding touching public goods, strangers, keeping a distance from “patients,” avoiding densely populated places and public transport (Rubin et al., 2009), or even resigning from jobs that are perceived to be dangerous (Yin and Ni, 2021).

##### Substitution Behavior (E.g. Using Technologies)

Service provision based on digital and artificial intelligence technology has become a possible solution to replace human service provision (Nayal et al., 2021), triggering changes in consumer behavior by using technology-mediated services (such as robots) to replace manual services (Kim et al., 2021).

#### Negative Resistance or Disruptive Behaviors

##### Resisting Behavior

People with low economic status are more likely to be vigilant about the public health information they receive are less willing to take recommended safety measures and may be more susceptible to “fake news” (Van Bavel et al., 2020). Misunderstandings and worries about the pandemic may also cause the public to refuse to comply with preventive measures (Prati et al., 2011). When people are less worried about the pandemic, they are less likely to engage in hygiene behaviors (such as washing hands), comply with social distance regulations, or be vaccinated if vaccines are available (Taylor, 2019). People also resist or refuse to participate in protective actions proposed by the government when they maintain an optimistic bias about the consequences of the outbreak (Fragkaki et al., 2021).

##### Panic Consumption Behavior

During the pandemic, a large number of customers stocked up on daily necessities to avoid the expected future threat due to uncertainty and panic arising from perceived scarcity, resulting in panic buying (Omar et al., 2021). People flooded hospitals and clinics unnecessarily when they misunderstood their minor illness as a sign of a serious infection (Asmundson and Taylor, 2020a, 2020b).

##### Negative Idleness or Sabotage Behavior

Anxiety is an important driving force of behavior (Taylor, 2019). Overly anxious individuals may engage in socially disruptive behaviors, especially for frontline service workers who are directly exposed to the outbreak (e.g., hotel staff), and may result in negative idleness (e.g., tardiness and absenteeism) or even disruptive behaviors or sabotage (Karatepe et al., 2021).

##### Excessive Stress Behavior

Anxiety and depression caused by the economic difficulties and employment difficulties caused by the crisis may result in various excessive stress behaviors, such as alcoholism (Ahmed et al., 2020) drug abuse (Nagelhout et al., 2017), even suicide (Milner et al., 2013), etc.

### The Boundary Conditions

#### Sociodemographic Factors

The impact of economic or employment difficulties caused by the pandemic on mental health may be related to socio-demographic factors, including age, gender, ethnicity, family size, occupation, and income (Ferry et al., 2021). Age is one factor. Young people are more likely to have a higher level of anxiety and stress due to the pandemic and corresponding intervention measures than the elderly (Mann et al., 2020; Salameh et al., 2020; Hu and Qian, 2021; Ribeiro et al., 2021). Young people with mental health problems are especially likely to experience adverse health, well-being, and employment outcomes with long-term consequences (Bauer et al., 2021). However, there are also arguments that the elderly may have greater financial difficulties due to the increase in medical expenses during the epidemic, which may trigger mental health problems (Van Bavel et al., 2020), and the elderly’s negative health consequences have been long-term ones (Van Bavel et al., 2020).

Gender is another one. Studies have shown that women are more likely to have higher levels of anxiety and stress when faced with possible physical health problems (Salameh et al., 2020; Ferry et al., 2021; Ribeiro et al., 2021). However, when there is the fear of losing their job and the economic anxiety surrounding this possibility, the psychological distress level is more serious for male than female employees (Timming et al., 2021). The third factor is ethnicity. Black and ethnic minority respondents have a higher level of economic anxiety (Mann et al., 2020). The study by Timming et al. (2021) shows that, compared with non-Hispanic respondents, Hispanic respondents are significantly more anxious about losing their jobs. The fourth factor is family size and the number of children. Respondents from families with no children have lower levels of economic anxiety (Mann et al., 2020). People living non-marital life have higher levels of psychological distress (Ferry et al., 2021).

Occupation is the fifth factor. People working at the emergency and customer-facing services, such as doctors, medical staff, police forces, frontline volunteer organizations, and bankers, have a higher risk of infection and subsequent mental stress (Shammi et al., 2020). The mental health of the unemployed, self-employed, and private professionals is worse than that of government professionals (Ali et al., 2020) for increased income (or economic) uncertainty caused by the pandemic (Patel and Rietveld, 2020) or for self-isolation or social distancing measures (Auerbach and Miller, 2020).

The sixth factor is income status. Most studies show that economic hardship resulting from the pandemic may make those disadvantaged groups (e.g., those living in poverty, low-income families, homeless, and refugees) the most vulnerable to experience the corresponding negative consequences (Van Bavel et al., 2020; Długosz, 2021; Hu and Qian, 2021). The mental health of people with disabilities and chronic diseases (Pettinicchio et al., 2021), living alone, and socially marginalized people is even more hostile (Kwong et al., 2020). However, some studies have suggested that the pandemic has a greater impact on the mental health of employees from high-income families (Ferry et al., 2021).

#### Personality Traits and Psychological Conditions

Personality traits and psychological conditions play an important role in the formation of mental health. Fisher et al. (2021) suggested that depressed and anxious psychological states during the epidemic were associated with diminished energy, functional efficiency, optimism, creativity, engagement, and the ability to focus and solve problems, all of which are necessary for social and economic participation. During the pandemic, those with low collective self-esteem, low responsibility, and low openness to experience have higher levels of economic anxiety, as do those with high levels of neuroticism, perceived vulnerability to illness, and attribution from large group activities (Mann et al., 2020). People with mental and physical health conditions may have higher levels of depression and anxiety because they are more likely to be unemployed and are prone to have higher levels of depression and anxiety (Hao et al., 2020; Kwong et al., 2020; Jung et al., 2021). Extreme loneliness is the main cause of psychological distress (Mikocka-Walus et al., 2021).

Emotional responses are part of the behavioral immune system. McKay et al. (2020) suggested that emotional reactions such as aversive tendencies and sensitivities are moderators of people’s disease sensitivity and anxiety. High perceived risks, especially economic risks, are significantly associated with less positive emotions and more negative emotions, leading to more severe mental health problems (Han et al., 2021). The “optimism bias” may help individuals to avoid negative emotions (Van Bavel et al., 2020); however, it may not be conducive for people to engage in behavior change in response to non-pharmacological interventions while individuals with high levels of anxiety and high perceived severity are more likely to be involved in behavior change (Fragkaki et al., 2021).

#### External Environment

The complex factors of population density, health care capacity, limited resources and existing poverty, environmental factors, social structure, cultural norms, the number of people already infected, and the rapidly occurring community transmission of COVID-19 virus in a country or region can all contribute to public fears, which may lead to higher levels of mental health problems (Shammi et al., 2020).

##### Level of Economic Development or Socio-Economic Crisis

People in low- and middle-income countries may have higher levels of stress, anxiety, and depression than those in high- and middle-income countries (Tee et al., 2021). In lower-middle-income countries with socio-economic crises, political instability, dense population and limited resources, the stress and anxiety during the pandemic are high (Salameh et al., 2020). Even in high-income countries such as Canada and the United Kingdom, deterioration in mental health has been reported, and are increasing along with the extension of the pandemic period (Zajacova et al., 2020).

##### Government Economic Intervention Policies or Welfare Policies

Policies that reduce economic stress (e.g., economic interventions such as emergency response benefits) may alleviate the level of mental health deterioration in the early stages of a pandemic by reducing economic hardship and making people less worried about their economic situation (Zajacova et al., 2020). Vaccine-based interventions help to mitigate the economic impact of the outbreak (Meltzer et al., 1999).

## Future Research Directions

### Mental Health Management

#### Monitoring and Preventive Measures

For policymakers, health authorities and health care professionals, it is very important to understand the impact of health anxiety on behavior. Future research should investigate the monitoring and preventive measures for different industries or different groups so as to help the government, service providers and employers understand the groups that should be given priority in mental health support (Ferry et al., 2021) and better conduct mental health rehabilitation. More studies are needed to examine the risk assessment of the pandemic, reliable risk communication with risk groups, the establishment of a cross-departmental management task force, and other measures.

#### Social Protection Measures and Relief Programs

Social protection measures include daily demand provision and social support (Jung et al., 2021) and cash transfer programs (Bauer et al., 2021). Future research should examine how to effectively use social protection measures (or relief plans) to solve the short-term and long-term effects of economic uncertainty caused by large-scale epidemics or economic crises on mental health. First, it is necessary to study how to support individual and family cash transfer programs to support young people’s future life opportunities and break the vicious circle between mental illness and poverty that puts many young people at a disadvantage in socio-economic and mental health (Bauer et al., 2021). Second, it is essential to study the physical and mental health of the most economically disadvantaged during economic downturns (Holmes et al., 2020; Bierman et al., 2021), and specialized relief measures that target low-income populations (Shammi et al., 2020). Third, future research should consider both material and social supports in the examination of social protection measures (or relief programs). Fourth, future research attention needs to be paid to employee assistance programs, with a particular focus on mental health support for male employees.

#### Intervention and Rehabilitation Measures

Interventions to reduce economic uncertainty and economic risks should be a focus of future research from two aspects. Future research can be conducted around three aspects: First, to examine how to cultivate an individual’s adaptive mentality to epidemics. Second, to explore individual resilience and psychological rehabilitation during and after a pandemic crisis (Hjemdal et al., 2011). Third, to explore the use of online interaction for social and mental health support. During the pandemic, providing remote mental health services is very important (Salameh et al., 2020). Future studies should examine online interactions to cultivate empathy and a sense of connection to enhance mental health (Schroeder et al., 2017; Waytz and Gray, 2018).

### Consequences of Mental Health

There are currently few studies on the behavioral consequences of mental health, and more research is needed to understand the behavioral consequences of mental health caused by the epidemic. For example, the current research mainly focuses on panic buying behavior, and other compensatory behaviors can be added in the future, such as increasing the number of purchased goods, increasing specific food consumption, online shopping, and so on. Another example is to understand how individual factors (including health anxiety) specifically affect people’s behavior in response to the pandemic (Asmundson and Taylor, 2020b). In addition, more research is required to examine the impact of the economic impact of the epidemic on the long-term behavior of individuals, especially stressful behaviors such as alcohol abuse, drug abuse, and suicide.

### Impact of Macro-Environmental Factors

#### Culture

In different cultural contexts (e.g., collectivism vs. individualism), economic distress and non-interventional measures such as social distancing may have different effects on mental health. From an evolutionary psychology perspective, when a group encounters a collective threat, strict rules may help the collective to coordinate and survive (Roos et al., 2015). In the face of a pandemic, a culture that is accustomed to putting freedom above safety can make community coordination difficult. However, currently there is little comparative research on mental health and behavior changes specifically for different cultures, and it is worthy of further thinking in the future.

#### Ethnic Group

People of different ethnic groups may have different attitudes and behaviors toward the epidemic. Further research is needed to examine the different responses of different ethnic groups to the epidemic (Rubin et al., 2009). Moreover, ethnic groups may have different degrees of xenophobia due to fear of coronavirus, and more research is needed to understand the relationship between coronavirus phobia and coronavirus-related xenophobia, and the possible role of individual difference variables (e.g., susceptibility to disease) within an ethnic group (Taylor, 2019).

#### Economic Development

Future research may examine the relationships between economic development and the impact of the pandemic on mental health based on the economic status of different countries, and explore solutions to the severe psychosocial health phenomena that may be caused by socio-economic crises in economically underdeveloped countries amid a large scale crisis.

#### Country

Relatively little research is focused on how psychological distress caused by the pandemic varies across countries. Future studies can compare and analyze the differences in the level of psychological distress in different countries with different economic conditions. As countries have achieved varying degrees of success in controlling the spread of the COVID-19 virus (Patel and Rietveld, 2020). Future research based on international data can further explore the level of psychological distress in countries where government interventions are relatively successful, in comparison with those countries that are not so successful.

### Long-Term Effects

As the COVID-19 pandemic continues to evolve, the sources of psychological distress surrounding the pandemic and the degree of psychological distress may change (Piltch-Loeb et al., 2021). The extant research mainly focuses on the early or short-term psychological impact of the pandemic. Long-term longitudinal research should be added in the future to investigate the sources of psychological and mental distress at different time points (Magnavita et al., 2020). Although a large number of studies have found a positive relationship between the economic uncertainty (or difficulties) and mental health problems, other studies do not degree with the relationship between deteriorating mental health and the level of job insecurity and financial impact (Kwong et al., 2020). Further empirical research is needed to understand the interrelationships among various antecedents and how different factors mediate or moderate the relationship between the pandemic and mental health.

## Conclusion

This conceptual analysis article explores two mechanisms (i.e., economic distress and employment distress) that lead to the deterioration of individuals’ mental health. The proposed conceptual framework explains how the COVID-19 pandemic and public health interventions affect people’s mental health, the responding coping behaviors. The extant literature provides evidence supporting the hypothesis that the COVID-19 pandemic and its associated measures increase individual economic uncertainty and employment uncertainty, thereby triggering mental health problems and coping behaviors. The findings of most studies support this mechanism from the onset of the pandemic to the emergence of economic distress and employment distress, to the deterioration of mental health, and then to changes in people’s behaviors. Supportive evidence was found in different countries (e.g., the United States, China, Bangladesh, Italy, etc.) and in different groups (elderly, young, disabled, mentally ill, etc.).

## Author Contributions

XL: conceptualization, methodology, and writing – original draft preparation. ZL: conceptualization and writing – reviewing and editing. All authors contributed to the article and approved the submitted version.

## Funding

This research was supported by the Educational Commission of Fujian Province of China (grant no. JAS20129) and the Science Foundation of Jimei University, China.

## Conflict of Interest

The authors declare that the research was conducted in the absence of any commercial or financial relationships that could be construed as a potential conflict of interest.

## Publisher’s Note

All claims expressed in this article are solely those of the authors and do not necessarily represent those of their affiliated organizations, or those of the publisher, the editors and the reviewers. Any product that may be evaluated in this article, or claim that may be made by its manufacturer, is not guaranteed or endorsed by the publisher.
